# Transcriptomic and metabolic responses of *Calotropis procera* to salt and drought stress

**DOI:** 10.1186/s12870-017-1155-7

**Published:** 2017-12-04

**Authors:** Mohammed Z. Mutwakil, Nahid H. Hajrah, Ahmed Atef, Sherif Edris, Mernan J. Sabir, Areej K. Al-Ghamdi, Meshaal J. S. M. Sabir, Charlotte Nelson, Rania M. Makki, Hani M. Ali, Fotouh M. El-Domyati, Abdulrahman S. M. Al-Hajar, Yoann Gloaguen, Hassan S. Al-Zahrani, Jamal S. M. Sabir, Robert K. Jansen, Ahmed Bahieldin, Neil Hall

**Affiliations:** 1 Biotechnology Research Group, Department of Biological Sciences, Faculty of Science, King Abdulaziz University (KAU), P.O. Box 80141, Jeddah, 21589 Saudi Arabia; 2 Department of Genetics, Faculty of Agriculture, Ain Shams University, Cairo, Egypt; 3 Princess Al-Jawhara Al-Brahim Centre of Excellence in Research of Hereditary Disorders (PACER-HD), Faculty of Medicine, King Abdulaziz University (KAU), Jeddah, Saudi Arabia; 4 Centre for Genomic Research, The University of Liverpool, Liverpool, L170AH UK; 5 The Earlham Institute, Norwich Research Park, Norwich, NR4 7UH UK; 6 College of MVLS, Glasgow Polyomics, University of Glasgow, Glasgow, UK; 7 Department of Integrative Biology, University of Texas at Austin, Austin, TX 78712 USA

**Keywords:** Salt stress, Drought stress, Transcriptomics, Metabolomics, Myo-inositol

## Abstract

**Background:**

*Calotropis procera* is a wild plant species in the family Apocynaceae that is able to grow in harsh, arid and heat stressed conditions. Understanding how this highly adapted plant persists in harsh environments should inform future efforts to improve the hardiness of crop and forage plant species. To study the plant response to droμght and osmotic stress, we treated plants with polyethylene glycol and NaCl and carried out transcriptomic and metabolomics measurements across a time-course of five days.

**Results:**

We identified a highly dynamic transcriptional response across the time-course including dramatic changes in inositol signaling, stress response genes and cytokinins. The resulting metabolome changes also involved sharp increases of myo-inositol, a key signaling molecule and elevated amino acid metabolites at later times.

**Conclusions:**

The data generated here provide a first glimpse at the expressed genome *of C. procera,* a plant that is exceptionally well adapted to arid environments. We demonstrate, through transcriptome and metabolome analysis that myo-inositol signaling is strongly induced in response to drought and salt stress and that there is elevation of amino acid concentrations after prolonged osmotic stress. This work should lay the foundations of future studies in adaptation to arid environments.

**Electronic supplementary material:**

The online version of this article (10.1186/s12870-017-1155-7) contains supplementary material, which is available to authorized users.

## Background

*Calotropis procera* is a flowering plant in the family Apocynaceae (Geneianales). It is native to North Africa, Tropical Africa, Western Asia, South Asia, and Indochina. The plant contains a milky sap consisting of a complex mix of chemicals including “cardiac aglycones” belonging to the same chemical family as therapeutic chemicals found in foxgloves (*Digitalis purpurea*), a related family in the asteroid I clade. It is a desert plant that is commonly known as Ushar or Madar. The medicinal use of this species was known to ancient Egyptians [1]. Its traditional medicinal uses include the treatment of leprosy [2], muscular spasms, dysentery, fever, rheumatism, asthma and as an expectorant and purgative [3–6]. In addition, scientific evidence has shown that *C. procera* possesses anticancer activity [7] antibacterial [8], nematocidal [9] and larvicidal activities [10–12]. Ecophysiological studies of *C. procera* have indicated that the photosynthetic capacity of the leaves can change with water availability due to changes in both stomatal and non-stomatal factors. However, the photosynthetic capacity is still relatively high during dry periods compared to ‘wet’ periods and no chronic photoinhibition occurs during dry periods [13] demonstrating that *C. procera* has a high level of tolerance to drought stress and high light.

Globally, the greatest uncontrolled factor in crop production is the unpredictability of water availability. To achieve sustainable crop production for the future, new crop varieties that have improved water use efficiency will have to be developed. Water use efficiency is given by the ratio of net carbon assimilation in photosynthesis and the water lost by transpiration. Consequently, the ability to regulate water lost by transpiration and maximize photosynthetic carbon assimilation are both potentially important factors when attempting to improve a crop’s water use efficiency. Clearly, *C. procera* has been shown to withstand arid conditions and maintain high rates of photosynthesis. Photosynthesis is a major determinant of crop growth and productivity, as well as being a crucial parameter in determining the distribution of species and, at an ecosystem level, being the major route by which the biosphere affects the composition of the atmosphere, with all the implications that has for global climate change. Optimizing the rate of photosynthesis will be crucial in maximizing crop productivity and bio-fuel production in arid regions. Understanding the molecular mechanisms by which *C. procera* tolerates drought successfully will offer a unique insight into how we may modify crops for improved drought tolerance and productivity.

Recent studies have utilized next generation sequencing for molecular discovery in *C. procera*. RNA-seq approaches have been used to identify a *Usp*-like gene that is putatively involved in response to heat and/or drought stress [14]. While Pandey et al. [15] have used both transcriptomic and metabolomic approaches to study the glycoside biosynthetic pathway, which is valuable for the production of potential pharmacological compounds. Ramadan et al. [16] have used metabolomics and lipidomics to study the response to water stress and concluded that the plant has a very rapid response to water availability and water loss, which is a likely adaptation to its harsh natural environment.

In all plant systems studied thus far the phytohormone abscisic acid (ABA) has been shown to function as a key regulator of the response to drought and salinity and has a pivotal function as a growth inhibitor [17, 18]. ABA activates reprogramming of cellular mechanisms of abiotic stress adaptation and also carbohydrate and lipid metabolism [19]. Lipids are also thought to be intimately involved in signaling during plant drought and salt stress. Levels of phosphatidylinositol bisphosphate, phosphatidic acid, and DGPP diacylglycerolpyrophosphate are upregulated upon salt stress [20]. The inositol phosphate myo-inositol hexakisphosphate also has a role as a signaling molecule regulating stomatal closure by triggering the release of calcium from endomembrane stores [21].

In this study, we compare the transcriptome and metabolome responses of the *C. procera* plants exposed to polyethylene glycol (PEG), as a non-absorbed osmoticum often used to assess drought stress, and to osmotic stress induced by salt (NaCl). We demonstrate there are distinct genome-wide responses, which are highly dynamic over the observed time course of treatment. We also integrate this with metabolomics data to compare the differences in accumulated metabolites through the time course. Our data identified large changes in regulation of cytokinins and suggests a key role of inositol signaling in both the transcriptomic and metabolomic profiles. Physiological changes such as the accumulation of amino acid metabolites also appear to be associated with osmotic stress. We discuss how these findings could be further studied to understand adaptation mechanisms to extreme aridity.

## Results and discussion

### Sequence generation and transcript assembly

**Fig. 1:**
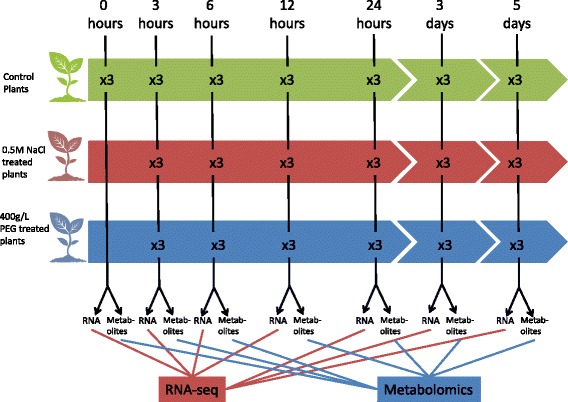
Experimental design for this study. Three groups of plants were used. A control group that were administered water, plants that were administered 0.5 M NaCl and a group administered 400 g/L of PEG at time 0. They were followed for 5 days and three plants samples at each time point. Metabolites and RNA were extracted from 3 replicates from each group and the data compared

**Fig. 2:**
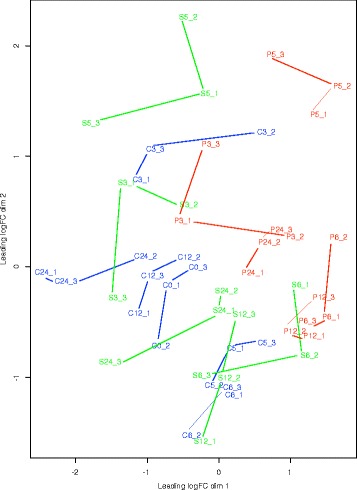
Multi-Dimensional Scaling plot for count data. MDS plot based on raw read count data of 20,000 genes for each sample. C6, C12, C24, C3 and C5 correspond to control plants at 6 h, 12 h, 24 h, 3 days, and 5 days, respectively. S6, S12, S24, S3 and S5 correspond to salt treated plants at 6 h, 12 h, 24 h, 3 days, and 5 days, respectively. P6, P12, P24, P3 and P5 correspond to PEG treated plants at 6 h, 12 h, 24 h, 3 days, and 5 days, respectively. Each replicate is labelled _1, _2, or _3. Lines connect samples from the same treatment and time point

The experimental design for the study is shown in Fig. 1. Each sequence library generated a minimum of 16 million reads with the majority of samples producing in excess of 20 million reads. The number of reads generated for each sample is shown in Additional file 1: Table S1. These were combined and assembled into transcripts to give 187,923 transcripts in total with an average length of 1049 nt. Approximately 85% of all reads were included in the final assembly. We assume the remaining reads were either low quality reads or very weakly expressed transcripts which did not form contigs as no major contaminants were identified. The assemblies were annotated using BLAST2GO [22] resulting in 52,108 annotated transcripts of which 28,265 received informative GO terms. Clustering of the all samples based on 20,000 most highly expressed genes using a multidimensional scaling plot based on leading log2 fold changes demonstrates that there is weak clustering replicates however the control samples, and the different treatments form distinct but overlapping clusters (Fig. 2). The fact that discrete clusters are not formed may not be unexpected given the that these are field samples and not cultivated plants which may have substantial genetic diversity, also other processes like circadian regulation of genes will affect sample clustering. From the clustering of the samples demonstrates that circadian effects don’t have a major impact on the data 24 h, 3 days and 5 days do not lie closer to 0 h than 6 and 12 h. There is also good separation between the controls and treated plants at equivalent time points. We also observe that dim 1 (x-axis) separates control from treated samples fairly well, while dim2 (y-axis) separates the treated time points up to 24 h from the 3 and 5 day treatments. There is an unusual pattern of expression at day 3 as the controls are perturbed from the other control samples more than salt and PEG treatments at day 3. This might also explain why day 3 shows fewer apparently differentially expressed genes than 24 h and 5 days.

To control for any circadian regulated genes each the gene expression in each group of plants was compared to control plants sampled at the same time point. Therefore, genes that are changing in expression due to circadian regulation alone should have significantly different sequence read counts between the control and treated samples. Additional file 2 shows the annotation, relative fold-changes and *P*-values for each gene assembly which was significantly regulated between conditions.

### Differential response to PEG and NaCl stress

**Fig. 3:**
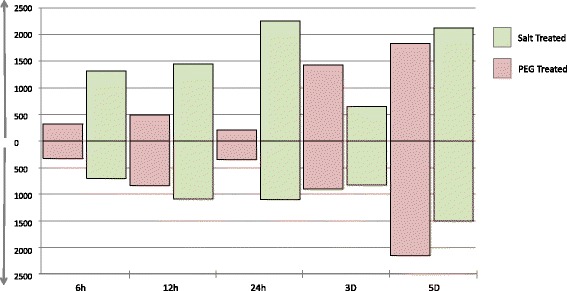
Number of up and downregulated genes in PEG and NaCl treated *C. procera* plants. Bars above the x-axis represent the number of up regulated genes relative to the control plants sampled at the same time point and bars below the x-axis represent the number of down regulated genes relative to the control plants sampled at the same time point

The number of genes regulated in response to NaCl treatment was much higher than that regulated in response to PEG treatment in the first 24 h (Fig. 3). After three days, the number of genes is reduced for NaCl treated plants compared to that of PEG treated plants although as mentioned above, this time-point appears to have an unusual profile (Fig. 2) in the control plants compared to the other time-points. And at 5 days the number of regulated genes are largely similar (Fig. 3).

**Fig. 4:**
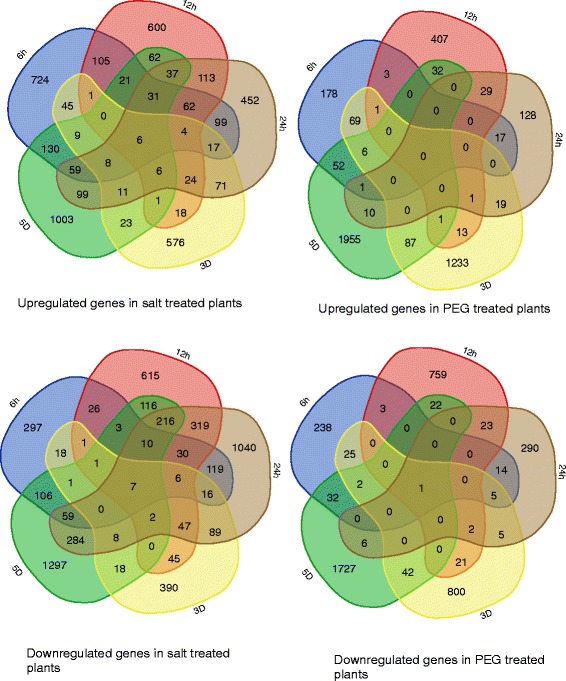
Venn diagrams of shared up and downregulated genes in *C. procera* across time. Each group is labelled 6 h, 12 h, 24 h, 3D, 5D for 6 h, 12 h, 24 h, 3 days, and 5 days, respectively

Expression was also compared between time-points and between treatments. Comparisons are shown in Fig. 4 and Additional file 3: Figure S1. There were very few regulated genes across all time points in either the NaCl or PEG treated plants suggesting that the response in the plant was very dynamic and rapidly changing. Comparing the number of shared regulated genes between the two treatments, it appears that the initial response due to the two different types of stresses is divergent as the overlap of shared genes is relatively small. However, the proportion of regulated genes shared between the treatments increases over time.

### Gene ontology enrichment

**Fig. 5:**
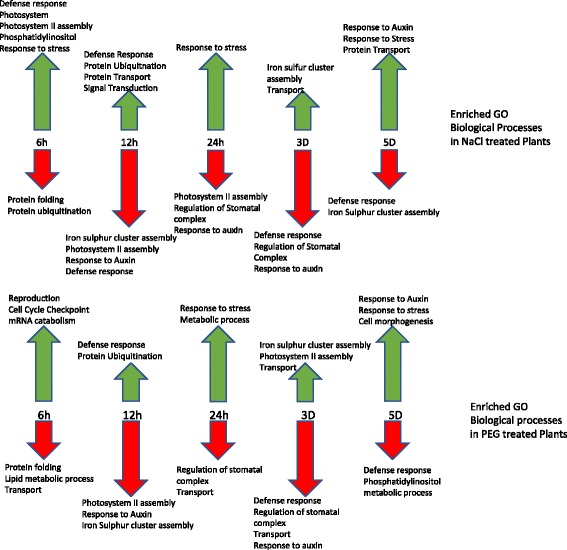
Selected Gene Ontology “Biological Process” terms in *C. procera* that are enriched in up and downregulated genes in PEG and NaCl treated plants. Enriched GO “Biological Processes” terms in NaCl treated plants, B. Enriched GO “Biological processes” terms in PEG treated plants. Up-arrows correspond to terms enriched in up regulated genes. Down-arrows correspond to terms enriched in down regulated genes

Figure 5 shows the results of the GO enrichment analysis “Biological Process” GO terms that are enriched for up and downregulated genes for each time point (a full list of enriched GO terms is shown in Additional file 4: Tables S3 and S4). At 6 h of salt treatment, the “Molecular Function” GO terms that are most upregulated are involved in signalling such as DNA binding, kinase and calcium binding (Additional file 4: Table S4).

### Metabolite analysis

**Fig. 6:**
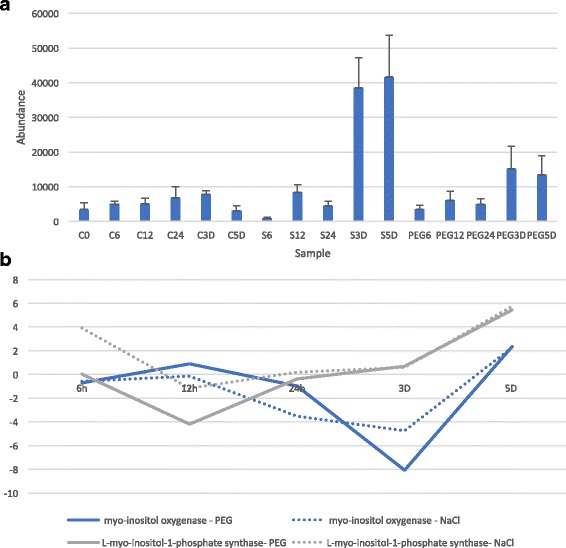
Myo-inositol abundance and myo-inositol metabolism gene expression in *C. procera*. **a**: Myo-inositol abundance as measured by metabolome analysis across all sample groups. **b**: Gene expression of myo-inositol metabolism genes myo-inositol oxygenase and L-myoinositol-1-phosphate synthase in PEG and NaCl treatments

**Fig. 7:**
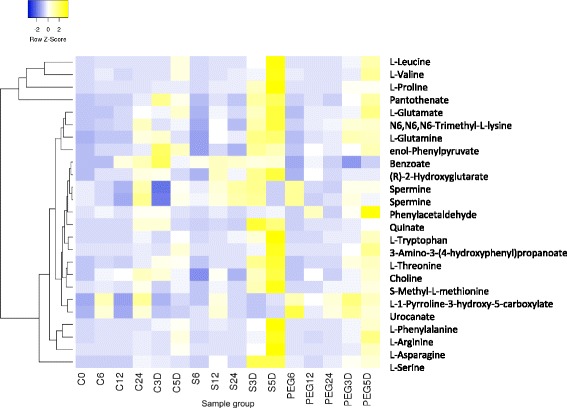
Amino acid pathway metabolite relative abundance across samples in *C. procera.* Heatmap of Z-scores for amino acid abundance as measured by metabolomics analysis across all sample groups. The dendrogram shows Average linkage clustering of metabolite abundance.

Out of 135 selected signals, 22 matched to authentic standards (out of 148 standard signals). shown in Additional file 1: Table S2. While the transcriptional response to both salt and PEG treatment appears in the first 12 h, the metabolic response was, as expected, more delayed. A key stress related compound that is identified as upregulated in our treatment samples is myo-Inositol. Figure 6 shows myo-inositol is accumulated in the salt treated samples and to a lesser extent in the PEG treated samples relative to the controls. Also, Amino acids and amino acid-related metabolites are observed at greater concentrations in salt treated samples (Fig. 7).

## Discussion

In the NaCl treated plants, there are only six upregulated and seven downregulated genes that were shared across time points and in the PEG treated plants, there was just one down-regulated gene (Fig. 4), an O-methyltransferase similar to AT5G53810 in Arabidopsis. Some methyltransferases have been demonstrated to be involved in the response to drought by reducing gibberellin levels [23]. While, similar genes in Brassica have been shown to be downregulated [24]. Since methyltransferases are regulated via a wide range of processes from flowering to lignin accumulation, the significance of this finding is difficult to interpret.

The annotated upregulated genes under salt stress include a xyloglucan specific endoglucanase, and putative calmodulin, peroxidase and lacase genes. Xyloglucan genes are believed to be regulated by abscisic acid [25] and can enable loosening of the cell walls by modifying xyloglucan. Cell wall loosening can become stiffened due to the action reactive oxygen species (ROS) generated by drought or salt stress [26].

Zhou et al. [27] have recently demonstrated that calmodulin genes *AtCaM1* and *AtCaM4* expression regulate NO_2_ a key signaling molecule in abiotic stress. The calmodulin gene identified here is most similar to *AtCaM3*, which is normally associated with response to heat stress [28] and suggests that these signaling pathways have diverged in *C. procera*.

The peroxidase gene, which was regulated across treatments, is similar to ATPXG2, a lipid body localized calcium dependent peroxidase, that can regulate guard cell membranes and thereby stomatal aperture during the drought response [29]. Therefore, it is likely that this protein is involved in closing the stomata aperture to prevent water loss during drought and salt stress.

The role of laccase in salt response is mysterious. This enzyme is a multi-copper glycoprotein oxidase able to oxidise a wide range of substrates including phenols and amines. Their precise physiological and biochemical role in higher plants remains largely unclear [30]. The identification of this gene as upregulated across the time course should be studied further to elucidate its role.

The downregulated genes across salt stress treatment include a cation antiporter, a peptide transporter, a phosphoribosyl transferase, protochlorophyllide reductase and fructose-1,6-bisphosphatase. Fructose-1,6-bisphosphatase has been demonstrated to be downregulated in tobacco during salt stress as a result of decreased chloroplast activity, which also affects protochlorophyllide reductase. This is consistent with observations in rice where it has been postulated that reduction in photosynthetic activity prevents the accumulation of photosensitive tetrapyrroles that can produce singlet oxygen molecules under stress conditions [31].

The downregulation of the cation antiporter is a surprising finding as it would be expected to be upregulated to help maintaining cytoplasmic homeostasis in response to increased extracellular NaCl levels, as has been reported in many other plants including wheat [32], grape [33], and Arabidopsis [34] and heterologous overexpression has also resulted in greater salt tolerance in many plant systems [35]. However, at six hours of salt treatment, this gene has eight-fold higher activity in the control plant. This unusual response may be associated with *Calotropis’* extreme hardiness. It is possible that the activity of this antiporter is replaced by an alternate protein, which is adapted to the high salt conditions. The gene is not significantly regulated in PEG treatments and, therefore, it is specific to osmotic stress associated with high NaCl levels.

### Gene ontology enrichment analysis

The pattern of enriched processes from the GO enrichment analysis is consistent with the plant rapidly adapting to stress as it senses an increase of extracellular ions. The PEG treated plants appear to respond much more slowly in the absence of osmotic pressure. At 12 h, the enriched biological processes are not associated with a stress response (Additional file 4: Table S3 and Figure 5). However, both treatment groups at 12 h have upregulation of defence response and ubiquitination processes suggesting protein turnover is occurring in response to stress (Additional file 4: Table S3 and Fig. 5).

The most downregulated gene measured (11 fold change in salt treated plants) is adenine phosphoribosyl transferase, a key metabolic enzyme participating in the cytokinin inactivation by phosphoribosylation. Cytokinin metabolic processes, including cytokinin biosynthesis, degradation, interconversion and inactivation have important regulatory roles in plant development [36, 37]. Analyses of Arabidopsis cytokinin biosynthesis and catabolism genes following NaCl treatment showed upregulation of biosynthesis for several hours before returning to pretreatment levels. Conversely, catabolic genes were initially repressed and followed by a gradual increase. Downregulation of phosphoribosyl transferase will increase cytokinin accumulation and potentially lead to a number of downstream effects including the upregulation xyloglucan endotransglucosylases/hydrolases [38, 39], which we also see upregulated throughout the salt treatment.

At 12 h (Additional file 4: Table S4), carbohydrate binding was identified as upregulated and enriched. Plants are known to synthesize carbohydrate-binding proteins upon exposure to stresses like drought, high salt, hormone treatment, pathogen attack [40]. This class of lectins is located in the cytoplasm and the nucleus. Different lectins are also seen to be downregulated at 24 h in the PEG treated plants (Additional file 4: Table S4). The role of these lectins is still unclear, however recent data from Arabidopsis suggests that some may act as transcriptional regulators in response to abiotic stress [41].

Early in the time course, the biological function ‘response to auxin’ was enriched in downregulated genes which reflects that the plants have slowed growth in response to stress (Additional file 4: Table S3). By day five, the plants have recovered and these genes were enriched in upregulated transcripts relative to the control plants in both treatments and auxin was promoting cell division and growth.

Enriched “Biological Process” GO terms in upregulated genes at six hours include phosphotidylinositol metabolic process (Additional file 4: Table S3 and Fig. 5). Phosphotidylinositol is involved in the generation of the second messengers, inositol 1,4,5-trisphosphate (IP_3_) and diacylglycerol (DAG). IP_3_ releases Ca^2+^ from internal stores, whereas DAG activates protein kinase C. It has been shown in Arabidopsis that IP_3_ levels rapidly increase in response to hyperosmotic stress [42, 43].

Iron-sulphur (Fe-S) cluster assembly appears to have complex regulatory pattern, being initially downregulated and then upregulated at 24 h and 3 days in both treatments, Iron-sulphur cluster assembly occurs in chloroplasts, mitochondria and cytosol and has been shown to be inhibited by heavy metal stress in rice [44]. Glutaredoxins, which reduce disulphide bridges and involved in iron-sulphur assembly and known to be involved in this process, are sensitive to oxidative stress and are downregulated in our experiment [45].

### Myo-inositol metabolism gene regulation is consistent with Myo-inositol abundance

Myo-inositol is a key intermediate in the inositol signaling pathway being a precursor of phosphotidylinositol and a potential entry-point into ascorbate generation [46]. It has been demonstrated in Arabidopsis that constitutive expression of myo-inositol 1-phosphate synthase 1, which is involved in myo-Inositol synthesis, protects against salt stress.

Our observation of myo-inositol from the metabolite data is consistent with the transcriptome data. As previously explained, we saw upregulation of phosphotidylinositol processes in the transcriptome of stressed plants. Also, we observed an increase in the expression of myo- inositol synthase expression peaking at nearly a 6-fold increase by day 5 (Fig. 6). Concurrently, we see a decrease in expression of myo-inositol oxygenase, an enzyme involved in the procession of myo-inositol into the ascorbic pathway and, therefore, acts to reduce myo-inositol [47]. The combined effect of the upregulation of myo-inositol synthesis and reduction in processing, will result in an accumulation of this important signaling molecule.

### Amino acids accumulation occurs in response to salt stress

It has been shown that an increase of amino acid concentration in *C. procera* was closely related to the rapid adjustment of water availability [16]. In other plants, it has also been demonstrated that amino acids such as proline, and amines such as glycine betaine and polyamines accumulate under drought stress act as osmolytes to maintain cell turgor and to stabilize cell proteins and structures during drought stress [48]. Proline acts as a systemic signal to promote stomatal closure in Arabidopsis, it is interesting to observe in *C. procera* that this is not triggered in the PEG treated plants but only in response to salt treatment (Fig. 7). As amino acids were highly represented in the identified metabolites their role, relative to other osmoprotectants can’t be quantified here and further studies are needed to asses this.

Polyphenols are found to be increasingly abundant in stressed samples, both PEG- and salt stress-induced show the response. Petunidin 3-O-glucoside, p-Coumaroylquinic acid and quinate levels are upregulated across time. It has been shown that phenolics are produced by plants mainly for protection against abiotic stress [49]. The role of these metabolites in abiotic stress is not well understood.

## Conclusions

The results of our analyses provide a first glimpse into the response of *C. procera* to salt and drought stress. Our results show that the plant undergoes complex changes in gene regulation across our five-day time course. The response to salt stress is initiated immediately as evidenced by the fact that at six hours key plant hormones such as cytokinin are being induced through the regulation of adenine phosphoribosyl transferase. These changes induce multiple downstream effects including effects including altering guard cell morphology to reduce water loss, reducing cell wall rigidity and accumulation of metabolites to balance osmotic pressure. It also appears from our study that inositol signaling also plays an important role in regulating stress response. Our approach of integrating metabolome and transcriptome analysis has enabled us to observe transcriptional changes giving rise to physiological changes in the plant, as with the accumulation of myo-inositol.

Understanding the plant’s adaptation to aridity is a vital step in producing crops that are able to cope with current rapid climate change. We argue that *C. procera* is a useful model for this study due to its remarkable ability to flourish in extremely harsh conditions. We speculate that this study will initiate future analysis of this system.

## Methods

### Stress treatment and leaf sampling

Seeds were collected from a field site near Jeddah, permission to collect plant material and preform fieldwork at the site was granted by the Governor of Makkah Province, Prince Khalid Al Faisal. All seeds in the experiment were collected from same fruit to reduce genetic heterogeneity. Seedlings were grown at a controlled temperature (25-28 °C) at 8000 lx light intensity for 16 h and an 8 h dark period. Seedlings were grown for one month after sowing. Three conditions were, then, applied across five time points (6 h, 12 h, 24 h, 3D and 5D) with three replicates in each case (Fig. 1). Careful leaf sampling was carried out in order to ensure for environmental conditions and variation between plants. Throughout the time course, samples were collected at regular intervals and sampling was taken from third leaf from top from each plant then with subsequent samples taken from the next lowest node at each time-point. All samples were flash frozen after collection in liquid Nitrogen (N2).

### RNA extraction

100 mg of frozen plant leaf material was ground with a pestle and mortar in liquid N2. This was used as input material for the RNeasy Plant Mini kit (B-Mecaptoethanol was added to the RLT lysis buffer) following the manufacturers protocol. RNA was eluted in 30 uL of nuclease-free water.

5 μg of RNA was DNase-treated using Ambion TURBO DNA-Free kit (cat no. AM1907). RNA was cleaned using 1.8× RNA Ampure beads and eluted in 20 uL of nuclease-free water. RNA was then run on the Agilent Bioanalyzer using an RNA Pico chip to check RNA integrity, and the concentration was measured by Qubit of which RIN values were all >7.

2 μg of RNA was RIbozero-depleted using the Illumina RIbozero Plant Leaf kit, and eluted in 12 uL. Ribozero-depleted RNA was, then, ran on the Agilent Bioanalyzer using an RNA pico chip. RNA was, then, used as input material for the Illumina ScriptSeq library preparation. After 13 cycles of PCR, the library was eluted in 20 uL of nuclease-free water. Finally, libraries were run on the Fragment Analyzer using the NGS High Sensitivity kit.

### Metabolite extraction

100 mg of the frozen plant tissue was homogenized in 2-mL Eppendorf tubes in a Retschmill. The metabolites were extracted from each aliquot in 1 mL of a homogenous mixture of −20 °C methanol: methyl-tert-butyl-ether: water (1:3:1), with shaking for 30 min at 4 °C, followed by another 10 min of incubation in an ice-cooled ultrasonication bath. Then, 650 μL of UPLC-grade methanol: water (1:3) was added, and the homogenate was vortexed and spun for 5 min at 4 °C in a table-top centrifuge. The addition of methanol: water leads to a phase separation, providing the upper organic phase, containing the lipids, a lower aqueous phase of which we focused on the aqueous metabolites. Metabolites were analysed by the University of Glasgow Polyomics facility using Orbitrap™ Q Exactive™ mass spectrometer with UltiMate™ 3000 RSLCnano separation system at the University of Glasgow Polyomics Facility and the resulting spectra analysed using the IDEOM software package.

### RNA sequencing

A number of 48 RNA samples were sequenced across three lanes of a HISEQ 2500 using 2 × 150 bp chemistry. The raw Fastq files were trimmed for the presence of Illumina adapter sequences using Cutadapt version 1.2. [50]. The option -O 3 was used, so the 3′ end of any reads, which match the adapter sequence for ≥ 3 bp, were trimmed. The reads were further trimmed using Sickle version 1.200 with a minimum window quality score of 20. Reads shorter than 10 bp after trimming were removed.

### Bioinformatic analysis

De novo assembly of the sequence reads was carried out using Trinity [51], which was also used to generate per-gene read counts. Further analysis was carried out in EdgeR [52] and DEseq. Tagwise dispersions were used to calculate differentially expressed genes with a false discovery rate of ≤ 0.05. Clustering was carried out using Cluster software package (version 3.0) and analysed using Heatmapper [53]. GO enrichment was performed using TopGO (https://bioconductor.org/packages/release/bioc/html/topGO.html) enriched terms were assigned weighted *P*-values using Fishers exact test.

## Additional files

Additional file 1: **Table S1.** Number of sequence reads generated for each sample. **Table S2.** List of metabolite signals matched to known standards. (DOCX 100 kb)

Additional file 2: Annotated regulated genes inferred from the RNAseq data. Description of the data: Genes identified as significantly regulated in PEG treated plants relative to control plants and genes identified as significantly regulated in Salt treated plants relative to control plants. For each gene (rows) normalized read counts and the resulting statistical analysis in EdgeR is presented. Also BLAST2GO annotations are given. (XLSX 4617 kb)

Additional file 3: Figure S1. Venn Diagrams showing the number shared and unique up and down regulated genes in each treatment at each time point. Up-regulated genes are on the left and down-regulated genes on the right. Blue circles denote the genes in the NaCl treated plants and red circles denote the genes in the PEG treated plants. (PDF 29 kb)

Additional file 4: Table S3. Enriched Biological GO terms in different treatments and time points. **Table S4** Enriched Molecular Function GO terms in different treatments and time points. (DOCX 130 kb)

## Abbreviations

ABA: Abscisic acid
BLAST: Basic Local Alignment Search Tool
bp: Base pair
D: Days
DAG: Diacylglycerol
DNA: Deoxyribonucleic acid
GO: Gene Ontology
h: Hours
IP_3_: Inositol 1,4,5-trisphosphate
Iron-Sulphur: Fe-S
mg: milligram
NaCl: Sodium Chloride
PCR: Polymerase Chain Reaction
PEG: Polyethylene glycol
RNA: Ribonucleic acid
ug: Microgram
uL: Microliter
