# Supplementary material for: Transcriptomic and metabolic responses of Calotropis procera to salt and drought stress
Source: BMC Plant Biol. 2017 Dec 4;17:231. doi: 10.1186/s12870-017-1155-7 (PMC5716246; doi:10.1186/s12870-017-1155-7)
Supplement: Supplementary file 1 — Table S1. Number of sequence reads generated for each sample. Table S2. List of metabolite signals matched to known standards. (DOCX 100 kb) [file 12870_2017_1155_MOESM1_ESM.docx]

**Table S1 Number of sequence reads generated for each sample**

| **Sample name** | **Treatment** | **TIME POINT** | Total Number of Reads |
| --- | --- | --- | --- |
| CL_C0_1 | CONTROL | 0h | 26894502 |
| CL_C0_2 |  |  | 22565182 |
| CL_C0_3 |  |  | 19923751 |
| CL_C6_1 |  | 6h | 21677685 |
| CL_C6_2 |  |  | 21456698 |
| CL_C6_3 |  |  | 19585543 |
| CL_C12_1 |  | 12h | 20113935 |
| CL_C12_2 |  |  | 19117568 |
| CL_C12_3 |  |  | 19468956 |
| CL_C24_1 |  | 24h | 40150435 |
| CL_C24_2 |  |  | 31489651 |
| CL_C24_3 |  |  | 37577498 |
| CL_C3D_1 |  | 3D | 19441070 |
| CL_C3D_2 |  |  | 27348763 |
| CL_C3D_3 |  |  | 31063750 |
| CL_C5D_1 |  | 5D | 34730917 |
| CL_C5D_2 |  |  | 19739929 |
| CL_C5D_3 |  |  | 16990528 |
| CL_S6_1 | SALT | 6h | 19315156 |
| CL_S6_2 |  |  | 21131581 |
| CL_S6_3 |  |  | 23676084 |
| CL_S12_1 |  | 12h | 18789854 |
| CL_S12_2 |  |  | 17137987 |
| CL_S12_3 |  |  | 16487599 |
| CL_S24_1 |  | 24h | 25158191 |
| CL_S24_2 |  |  | 20688249 |
| CL_S24_3 |  |  | 18701295 |
| CL_S3D_1 |  | 3D | 23177245 |
| CL_S3D_2 |  |  | 20625839 |
| CL_S3D_3 |  |  | 22656996 |
| CL_S5D_1 |  | 5D | 20885796 |
| CL_S5D_2 |  |  | 23922185 |
| CL_S5D_3 |  |  | 27288941 |
| CL_PEG6_1 | PEG | 6h | 30683851 |
| CL_PEG6_2 |  |  | 26389250 |
| CL_PEG6_3 |  |  | 26253327 |
| CL_PEG12_1 |  | 12h | 17980278 |
| CL_PEG12_2 |  |  | 18490844 |
| CL_PEG12_3 |  |  | 19284617 |
| CL_PEG24_1 |  | 24h | 20655630 |
| CL_PEG24_2 |  |  | 25277277 |
| CL_PEG24_3 |  |  | 20661051 |
| CL_PEG3D_1 |  | 3D | 27431177 |
| CL_PEG3D_2 |  |  | 24264886 |
| CL_PEG3D_3 |  |  | 25955579 |
| CL_PEG5D_1 |  | 5D | 13861354 |
| CL_PEG5D_2 |  |  | 33140378 |
| CL_PEG5D_3 |  |  | 24561520 |

**Table S2 List of metabolite signals matched to known standards**

| **MASS** | **RT** | **FORMULA** | **ISOMERS** | **PUTATIVE METABOLITE** | **CONFIDENCE** | **PVALUE** |
| --- | --- | --- | --- | --- | --- | --- |
| 117.0789701 | 11.65515077 | C5H11NO2 | 16 | L-Valine | 10 | 0.293 |
| 115.063346 | 12.07090964 | C5H9NO2 4 | 4 | L-Proline | 10 | 0.064 |
| 219.110724 | 8.643922873 | C9H17NO5 1 | 1 | Pantothenate | 10 | 0.545 |
| 192.0270172 | 14.30373387 | C6H8O7 | 12 | Citrate | 10 | 0.27 |
| 103.099721 | 18.51291524 | C5H13NO | 1 | Choline | 10 | 0.122 |
| 183.066151 | 12.88760869 | C5H14NO4P | 1 | Choline phosphate | 10 | 0.437 |
| 134.0215145 | 13.23455552 | C4H6O5 | 4 | (S)-Malate | 10 | 0.286 |
| 146.069075 | 13.28135 | C5H10N2O3 | 6 | L-Glutamine | 10 | 0.352 |
| 196.058279 | 12.30809693 | C6H12O7 | 11 | D-Gluconic acid | 10 | 0.216 |
| 105.042579 | 13.78381666 | C3H7NO3 | 3 | L-Serine | 10 | 0.294 |
| 342.1165012 | 13.37444834 | C12H22O11 | 42 | Sucrose | 10 | 0.391 |
| 267.0965389 | 9.390399993 | C10H13N5O4 | 3 | Adenosine | 10 | 0.439 |
| 119.0582951 | 13.00133974 | C4H9NO3 | 11 | L-Threonine | 10 | 0.199 |
| 135.0545172 | 9.847454285 | C5H5N5 | 1 | Adenine | 10 | 0.428 |
| 131.09463 | 11.07709053 | C6H13NO2 | 12 | L-Leucine | 10 | 0.174 |
| 174.1117078 | 21.11324582 | C6H14N4O2 | 2 | L-Arginine | 10 | 0.323 |
| 148.0371415 | 12.6605952 | C5H8O5 | 18 | (R)-2-Hydroxyglutarate | 8 | 0.241 |
| 1470530878 | 12.70040326 | C5H9NO4 | 14 | L-Glutamate | 10 | 0.74 |
| 165.078988 | 10.2479427 | C9H11NO2 | 7 | L-Phenylalanine | 10 | 0.017 |
| 132.053471 | 13.42537774 | C4H8N2O3 | 6 | L-Asparagine | 10 | ND |
| 204.0898525 | 11.49399701 | C9H11NO2 | 6 | L-Tryptophan | 10 | ND |
| 257.1026109 | 10.2479427 | C8H20NO6P | 1 | sn-glycero-3-Phosphocholine | 8 | ND |
