# Supplementary figures and images for: Transcriptomic and metabolic responses of Calotropis procera to salt and drought stress
Source: BMC Plant Biol. 2017 Dec 4;17:231. doi: 10.1186/s12870-017-1155-7 (PMC5716246; doi:10.1186/s12870-017-1155-7)

## Up Regulated

## Down Regulated

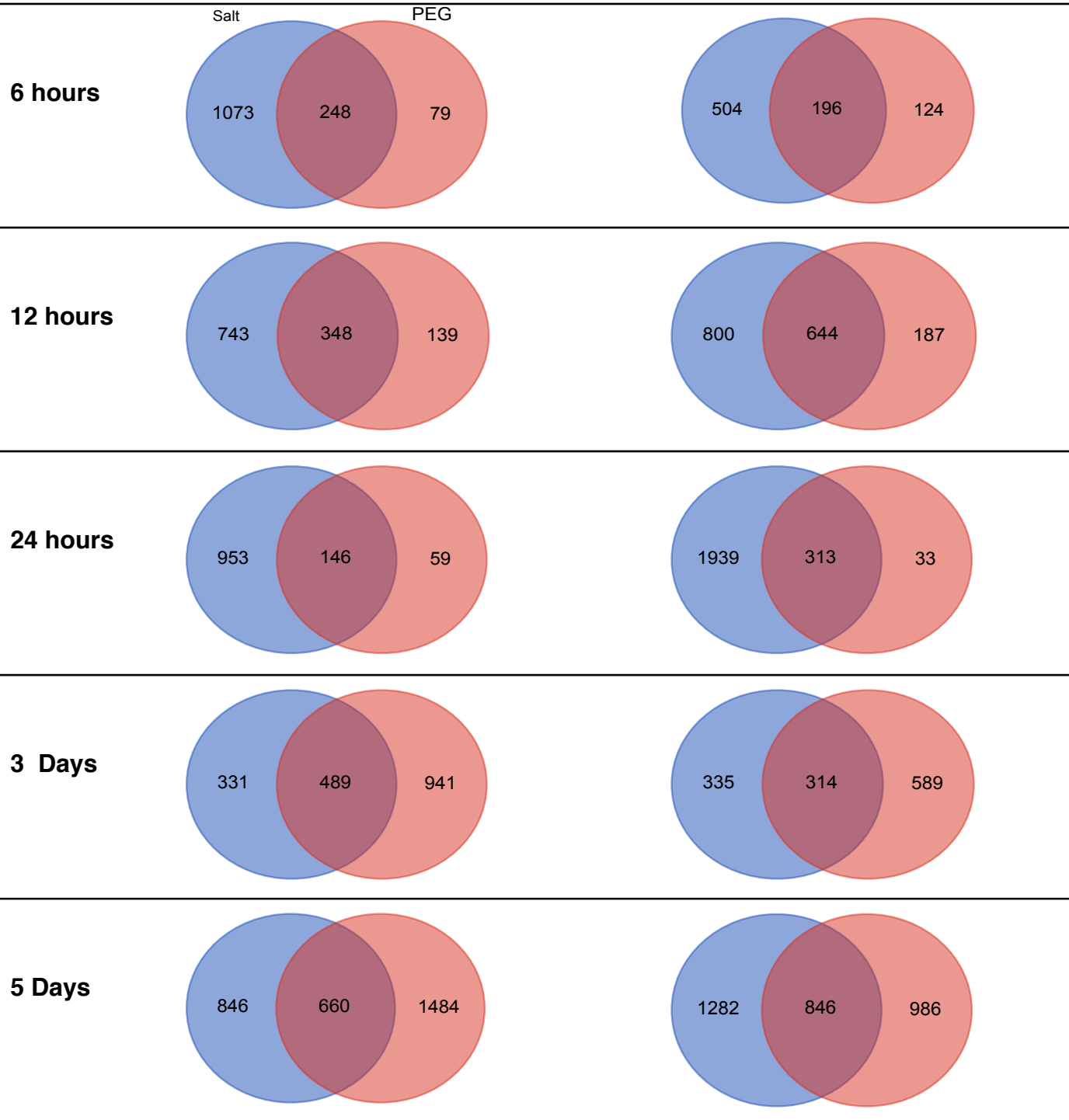

Supplement: Supplementary file 3 — Venn Diagrams showing the number shared and unique up and down regulated genes in each treatment at each time point. Up-regulated genes are on the left and down-regulated genes on the right. Blue circles denote the genes in the NaCl treated plants and red circles denote the genes in the PEG treated plants. (PDF 29 kb) [file 12870_2017_1155_MOESM3_ESM.pdf]
