# Supplementary material for: Transcriptomic and metabolic responses of Calotropis procera to salt and drought stress
Source: BMC Plant Biol. 2017 Dec 4;17:231. doi: 10.1186/s12870-017-1155-7 (PMC5716246; doi:10.1186/s12870-017-1155-7)
Supplement: Supplementary file 4 — Enriched Biological GO terms in different treatments and time points. Table S4 Enriched Molecular Function GO terms in different treatments and time points. (DOCX 130 kb) [file 12870_2017_1155_MOESM4_ESM.docx]

**Table S3 Enriched Biological GO terms in different treatments and time points.**

| Time-point | Up-regulated | | Down regulated | |
| --- | --- | --- | --- | --- |
|  | GO Annotation | P-value | GO Annotation | P-value |
| 6 hours  Salt  treatment | \| GO:0006952 \| defence response \| 7 \| 4 \| 0.19 \| 1 \| 8.40E-06 \| \| --- \| --- \| --- \| --- \| --- \| --- \| --- \| \| GO:0010207 \| photosystem II assembly \| 1 \| 1 \| 0.03 \| 4 \| 0.026 \| \| GO:0046488 \| phosphatidylinositol metabolic process \| 2 \| 1 \| 0.05 \| 8 \| 0.052 \| \| GO:0006950 \| response to stress \| 21 \| 5 \| 0.56 \| 2 \| 6.80E-05 \| \| GO:0015031 \| protein transport \| 10 \| 1 \| 0.26 \| 19 \| 0.238 \| | \| 8.40E-06 \| \| --- \| \| 0.026 \| \| 0.052 \| \|  \| \| 6.80E-05 \| \| 0.238 \| | \| GO:0006457 \| protein folding \| \| --- \| --- \| \| GO:0016567 \| protein ubiquitination \| \| GO:0006996 \| organelle organization \| \| GO:0009987 \| cellular process \| | \| 0.026 \| \| --- \| \| 0.09 \| \| 0.228 \| \| 0.247 \| |
| 12 hours  salt treatment | \| GO:0051026 \| chiasma assembly \| \| --- \| --- \| \| GO:0006952 \| defence response \| \| GO:0016567 \| protein ubiquitination \| \| GO:0051260 \| protein homooligomerization \| \| GO:0015031 \| protein transport \| \| GO:0019538 \| protein metabolic process \| \| GO:0007165 \| signal transduction \| \| GO:0000003 \| reproduction \| | \| 0.019 \| \| --- \| \| 0.124 \| \| 0.124 \| \| 0.156 \| \| 0.172 \| \| 0.094 \| \| 0.583 \| \| 0.156 \| | \| GO:0016226 \| iron-sulfur cluster assembly \| \| --- \| --- \| \| GO:0010207 \| photosystem II assembly \| \| GO:0009733 \| response to auxin \| \| GO:0006796 \| phosphate-containing compound metabolic ... \| \| GO:0006952 \| defense response \| | \| 0.0049 \| \| --- \| \| 0.0238 \| \| 0.0451 \| \| 0.1141 \| \| 0.1564 \| |
| 24 hours  salt  treatment | \| GO:0006950 \| \| response to stress \| \| \| --- \| --- \| --- \| --- \| \| GO:0044267 \| \| cellular protein metabolic process \| \| \|  \| \|  \| \| \|  \| \|  \| \| \|  \| \|  \| \| \|  \| \|  \| \| \|  \| \|  \| \| \|  \| \|  \| \| \|  \| \|  \| \| \|  \| \|  \| \| | \| 0.11 \| \| --- \| \| 0.15 \| | \| GO:0009567 \| double fertilization forming a zygote an... \| \| --- \| --- \| \| GO:0044767 \| single-organism developmental process \| \| GO:0009793 \| embryo development ending in seed dorman... \| \| GO:0010207 \| photosystem II assembly \| \| GO:2000123 \| positive regulation of stomatal  complex ... \| \| GO:0010154 \| fruit development \| \| GO:0006364 \| rRNA processing \| \| GO:0000902 \| cell morphogenesis \| \| GO:0009733 \| response to auxin \| | \| 0.0015 \| \| --- \| \| 0.0024 \| \| 0.0397 \| \| 0.0397 \| \| 0.0397 \| \| 0.073 \| \| 0.1147 \| \| 0.217 \| \| 0.4616 \| |
| 3 days  salt  treatment | \| GO:0016226 \| iron-sulfur cluster assembly \| \| --- \| --- \| \| GO:0006810 \| transport \| | \| 0.026 \| \| --- \| \| 0.21 \| | \| GO:0006952 \| defense response \| \| --- \| --- \| \| GO:2000123 \| positive regulation of stomatal complex ... \| \| GO:0009733 \| response to auxin \| \| GO:0007165 \| signal transduction \| | \| 0.0043 \| \| --- \| \| 0.0159 \| \| 0.0201 \| \| 0.5266 \| |
| 5 days  salt  treatment | \| GO:0009733 \| response to auxin \| \| --- \| --- \| \| GO:0006950 \| response to stress \| \| GO:0015031 \| protein transport \| \| GO:0006355 \| regulation of transcription, DNA-templat... \| | \| 0.036 \| \| \| --- \| --- \| \| 0.067 \| \| \| 0.195 \| \| \| 0.315 \| \| \|  \| \| | \| GO:0009567 \| double fertilization forming a zygote an... \| \| --- \| --- \| \| GO:0006952 \| defense response \| \| GO:0016226 \| iron-sulfur cluster assembly \| \| GO:0006996 \| organelle organization \| | \| 0.00063 \| \| --- \| \| 0.01235 \| \| 0.12609 \| \| 0.40674 \| |
|  |  |  |  |  |
| 6 hours  PEG  treatment | \| GO:0000003 \| reproduction \| \| --- \| --- \| \| GO:0000070 \| mitotic sister chromatid segregation \| \| GO:0000075 \| cell cycle checkpoint \| \| GO:0000184 \| nuclear-transcribed mRNA catabolic process \| | \| 0.83 \| \| --- \| \| 0.9 \| \| 0.18 \| \| 0.3 \| | \| GO:0006457 \| protein folding \| \| --- \| --- \| \| GO:0006629 \| lipid metabolic process \| \| GO:0006810 \| transport \| | \| 0.026 \| \| --- \| \| 0.114 \| \| 0.447 \| |
| 12 hours  PEG  Treatment | \| GO:0006952 \| defense response \| \| --- \| --- \| \| GO:0016567 \| protein ubiquitination \| | \| 0.037 \| \| --- \| \| 0.037 \|  \| 0.0031 \| \| --- \| \| 0.0138 \| \| 0.0319 \| \| 0.0319 \| \| 0.0436 \| \| 0.1499 \| \| 0.0382 \| \| 0.2776 \| \| 0.2776 \| \| 0.2905 \| \| 0.3674 \| \| 0.0348 \| \| 0.053 \| \| 0.5121 \| \| 0.3163 \| \| 0.0081 \| | \| GO:0010207 \| photosystem II assembly \| \| --- \| --- \| \| GO:0009733 \| response to auxin \| \| GO:0006796 \| phosphate-containing compound metabolic ... \| \| GO:0016226 \| iron-sulfur cluster assembly \| | \| 0.019 \| \| --- \| \| 0.028 \| \| 0.09 \| \|  \| |
| 24 hours PEG  Treatment | \| GO:0006950 \| response to stress \| \| --- \| --- \| \| GO:0044267 \| cellular protein metabolic process \| | \| 0.11 \| \| --- \| \| 0.15 \| | \| GO:2000123 \| positive regulation of stomatal complex ... \| \| --- \| --- \| \| GO:0006810 \| transport \| | \| 0.0079 \| \| --- \| \| 0.2984 \| |
| 3 days PEG  Treatment | \| GO:0016226 \| iron-sulfur cluster assembly \| \| --- \| --- \| \| GO:0010207 \| photosystem II assembly \| \| GO:0006810 \| transport \| | \| 0.0014 \| \| --- \| \| 0.0132 \| \| 0.4469 \| | \| GO:0006952 \| defense response \| \| --- \| --- \| \| GO:2000123 \| positive regulation of stomatal complex ... \| \| GO:0009733 \| response to auxin \| | \| 0.0043 \| \| --- \| \| 0.0159 \| \| 0.0201 \| |
| 5 days PEG  Treatment | \| GO:0009733 \| response to auxin \| \| --- \| --- \| \| GO:0006950 \| response to stress \| \| GO:0000902 \| cell morphogenesis \| | \| 0.0052 \| \| --- \| \| 0.0138 \| \| 0.1495 \| | \| GO:0006952 \| defense response \| \| --- \| --- \| \| GO:0046488 \| phosphatidylinositol metabolic process \| | \| 0.0043 \| \| --- \| \| 0.0315 \| |

**Table S4 Enriched Molecular Function GO terms in different treatments and time points.**

| Time-point | Up-regulated | | Down regulated | |
| --- | --- | --- | --- | --- |
|  | GO Annotation | P-value | GO Annotation | P-value |
| 6 hours  Salt  treatment | \| GO:0005509 \| calcium ion binding \| \| --- \| --- \| \| GO:0003677 \| DNA binding \| \| GO:0004812 \| aminoacyl-tRNA ligase activity \| \| GO:0016301 \| kinase activity \| \| GO:0016829 \| lyase activity \| \| GO:0005488 \| binding \| \| GO:0016747 \| transferase activity (acyl) \| | \| 4.10E-07 \| \| --- \| \| 0.0017 \| \| 0.0261 \| \| 0.2326 \| \| 0.4425 \| \| 0.0142 \| \| 0.6371 \| | \| GO:0046983 \| protein dimerization activity \| \| --- \| --- \| \| GO:0008236 \| serine-type peptidase activity \| \| GO:0003860 \| 3-hydroxyisobutyryl-CoA hydrolase \| \| GO:0005525 \| GTP binding \| \| GO:0016746 \| transferase activity (acyl ) \| \| GO:0005515 \| protein binding \| \| GO:0005488 \| binding \| \| GO:0016740 \| transferase activity \| | \| 0.0025 \| \| --- \| \| 0.003 \| \| 0.0191 \| \| 0.1678 \| \| 0.3797 \| \| 0.0108 \| \| 0.363 \| \| 0.4659 \| |
| 12 hours  salt treatment | \| GO:0030246 \| carbohydrate binding \| \| --- \| --- \| \| GO:0003677 \| DNA binding \| \| GO:0008236 \| serine-type peptidase activity \| \| GO:0016747 \| transferase activity \| \| GO:0016874 \| ligase activity \| \| GO:0016829 \| lyase activity \| \| GO:0016740 \| transferase activity \| | \| 0.10 \| \| --- \| \| 0.10 \| \| 0.17 \| \| 0.19 \| \| 0.36 \| \| 0.37  0.29 \| | \| GO:0008483 \| transaminase activity \| \| --- \| --- \| \| GO:0045439 \| isopenicillin-N epimerase activity \| \| GO:0016765 \| transferase activity (alkyl) \| \| GO:0005524 \| ATP binding \| \| GO:0046983 \| protein dimerization activity \| | \| 0.0061 \| \| --- \| \| 0.0122 \| \| 0.0598 \| \| 0.0756 \| \| 0.3108 \| |
| 24 hours  salt  treatment | \| GO:0008430 \| selenium binding \| \| --- \| --- \| \| GO:0030246 \| carbohydrate binding \| \| GO:0003676 \| nucleic acid binding \| \| GO:0008236 \| serine-type peptidase activity \| \| GO:0016491 \| oxidoreductase activity \| \| GO:0016757 \| transferase activity (glyco) \| \| GO:0016829 \| lyase activity \| \| GO:0016747 \| transferase activity (acyl) \| \| GO:0005509 \| calcium ion binding \| \| GO:0003677 \| DNA binding \| | \| 0.08 \| \| --- \| \| 0.08 \| \| 0.1 \| \| 0.14 \| \| 0.22 \| \| 0.26 \| \| 0.31 \| \| 0.47 \| \| 0.48 \| \| 0.11 \| | \| GO:0046983 \| protein dimerization activity \| \| --- \| --- \| \| GO:0005524 \| ATP binding \| \| GO:0004848 \| ureidoglycolate hydrolase activity \| \| GO:0045439 \| isopenicillin-N epimerase activity \| \| GO:0050281 \| serine-glyoxylate transaminase activity \| \| GO:0047672 \| anthranilate N-benzoyltransferase \| \| GO:0030246 \| carbohydrate binding \| \| GO:0003677 \| DNA binding \| \| GO:0008483 \| transaminase activity \| \| GO:0016410 \| N-acyltransferase activity \| \| GO:0051213 \| dioxygenase activity \| \| GO:0016788 \| hydrolase activity, on ester bond... \| \| GO:0003779 \| actin binding \| | \| 0.0016 \| \| --- \| \| 0.0095 \| \| 0.0298 \| \| 0.0298 \| \| 0.0298 \| \| 0.0587 \| \| 0.1405 \| \| 0.1812 \| \| 0.0336 \| \| 0.2615 \| \| 0.4033 \| \| 0.4432 \| \| 0.4878 \| |
| 3 days  salt  treatment | \| GO:0051213 \| dioxygenase activity \| \| --- \| --- \| \| GO:0016788 \| hydrolase activity, (ester bond) \| \| GO:0016765 \| transferase activity (alkyl) \| \| GO:0030246 \| carbohydrate binding \| \| GO:0051087 \| chaperone binding \| \| GO:0019904 \| protein domain specific binding \| \| GO:0008270 \| zinc ion binding \| \| GO:0016747 \| transferase activity (acyl) \| \| GO:0003723 \| RNA binding \| \| GO:0003824 \| catalytic activity \| \| GO:0016740 \| transferase activity \| \| GO:0016787 \| hydrolase activity \| | \| 0.011 \| \| --- \| \| 0.011 \| \| 0.047 \| \| 0.047 \| \| 0.056 \| \| 0.176 \| \| 0.26 \| \| 0.309 \| \| 0.438 \| \| 0.047 \| \| 0.233 \| \| 0.154 \| | \| GO:0016301 \| kinase activity \| \| --- \| --- \| \| GO:0003723 \| RNA binding \| \| GO:0046983 \| protein dimerization activity \| \| GO:0033926 \| glycopeptide alpha-N-acetylgalactosamini... \| \| GO:0016740 \| transferase activity \| | \| 0.0046 \| \| --- \| \| 0.0229 \| \| 0.0391 \| \| 0.1579 \| \| 0.048 \| |
| 5 days  salt  treatment | \| GO:0003676 \| nucleic acid binding \| \| --- \| --- \| \| GO:0004067 \| asparaginase activity \| \| GO:0004812 \| aminoacyl-tRNA ligase activity \| \| GO:0050638 \| taxadien-5-alpha-ol O-acetyltransferase ... \| \| GO:0046983 \| protein dimerization activity \| \| GO:0016874 \| ligase activity \| \| GO:0016746 \| transferase activity, ( acyl) ... \| \| GO:0030246 \| carbohydrate binding \| \| GO:0003682 \| chromatin binding \| \| GO:0016410 \| N-acyltransferase activity \| \| GO:0016757 \| transferase activity, (glyco) \| | \| 0.0069 \| \| --- \| \| 0.0314 \| \| 0.0314 \| \| 0.0314 \| \| 0.0654 \| \| 0.0261 \| \| 0.0170 \| \| 0.1475 \| \| 0.2003 \| \| 0.2736 \| \| 0.2905 \| \| 5 \| | \| GO:0008236 \| serine-type peptidase activity \| \| --- \| --- \| \| GO:0016884 \| carbon-nitrogen ligase activity \| \| GO:0016746 \| transferase activity, (acyl) .. \| \| GO:0004792 \| thiosulfate sulfurtransferase activity \| \| GO:0003860 \| 3-hydroxyisobutyryl-CoA hydrolase \| \| GO:0000062 \| fatty-acyl-CoA binding \| \| GO:0016765 \| transferase activity, (alkyl) \| \| GO:0051087 \| chaperone binding \| \| GO:0008080 \| N-acetyltransferase activity \| \| GO:0008270 \| zinc ion binding \| \| GO:0016874 \| ligase activity \| \| GO:0016740 \| transferase activity \| \| GO:0046872 \| metal ion binding \| \| GO:0016788 \| hydrolase activity, (ester bond) \| | \| 0.0006 \| \| --- \| \| 0.0024 \| \| 0.0028 \| \| 0.0207 \| \| 0.0410 \| \| 0.0610 \| \| 0.0996 \| \| 0.1183 \| \| 0.1546 \| \| 0.1819 \| \| 0.0085 \| \| 0.0048 \| \| 0.2264 \| \| 0.2781 \| |
|  |  |  |  |  |
| 6 hours  PEG  treatment | \| GO:0016788 \| hydrolase activity(ester bond) \| \| --- \| --- \| \| GO:0003723 \| RNA binding \| \| GO:0005509 \| calcium ion binding \| \| GO:0046872 \| metal ion binding \| \| GO:0003824 \| catalytic activity \| | \| 0.22 \| \| --- \| \| 0.25 \| \| 0.38 \| \| 0.38 \| \| 0.49 \| | \| GO:0046983 \| protein dimerization activity \| \| --- \| --- \| \| GO:0008236  GO:0016747 \| serine-type peptidase activity  transferase activity, (acyl) \| | \| 0.0049 \| \| --- \| \| 0.0331 \| \| 0.1334 \| |
| 12 hours  PEG  Treatment | \| GO:0016740 \| transferase activity \| \| --- \| --- \| \| GO:0008236 \| serine-type peptidase activity \| \| GO:0051213 \| dioxygenase activity \| \| GO:0046983 \| protein dimerization activity \| \| GO:0003677 \| DNA binding \| \| GO:0003824 \| catalytic activity \| \| GO:0005524 \| ATP binding \| | \| 0.01 \| \| --- \| \| 0.088 \| \| 0.159 \| \| 0.264 \| \| 0.48 \| \| 0.028 \| \| 0.573 \| | \| GO:0005524 \| ATP binding \| \| --- \| --- \| \| GO:0003779 \| actin binding \| \| GO:0016740 \| transferase activity \| \| GO:0005515 \| protein binding \| \| GO:0016787 \| hydrolase activity \| \| GO:0003824 \| catalytic activity \| | \| 0.03 \| \| --- \| \| 0.172 \| \| 0.387 \| \| 0.192 \| \| 0.494 \| \| 0.438 \| |
| 24 hours PEG  Treatment | \| GO:0008236 \| serine-type peptidase activity \| \| --- \| --- \| \| GO:0016787 \| hydrolase activity \| | \| 0.033 \| \| --- \| \| 0.069 \| | \| GO:0046983 \| protein dimerization activity \| \| --- \| --- \| \| GO:0016410 \| N-acyltransferase activity \| \| GO:0003779 \| actin binding \| \| GO:0016491 \| oxidoreductase activity \| | \| 0.00019 \| \| --- \| \| 0.04185 \| \| 0.09003 \| \| 0.20483 \| |
| 3 days PEG  Treatment | \| GO:0004792 \| thiosulfate sulfurtransferase activity \| \| --- \| --- \| \| GO:0004848 \| ureidoglycolate hydrolase activity \| \| GO:0005524 \| ATP binding \| \| GO:0016788 \| hydrolase activity, (ester bond) \| \| GO:0000062 \| fatty-acyl-CoA binding \| | \| 0.011 \| \| --- \| \| 0.011 \| \| 0.011 \| \| 0.017 \| \| 0.033 \| | \| GO:0003676 \| nucleic acid binding \| \| --- \| --- \| \| GO:0008568 \| microtubule-severing ATPase activity \| \| GO:0046983 \| protein dimerization activity \| \| GO:0016829 \| lyase activity \| \| GO:0097159 \| organic cyclic compound binding \| \| GO:1901363 \| heterocyclic compound binding \| \| GO:0005524 \| ATP binding \| | \| 0.0021 \| \| --- \| \| 0.0684 \| \| 0.0958 \| \| 0.3242 \| \| 0.0078 \| \| 0.0078 \| \| 0.4262 \| |
| 5 days PEG  Treatment | \| GO:0003677 \| DNA binding \| \| --- \| --- \| \| GO:0046983 \| protein dimerization activity \| \| GO:0004812 \| aminoacyl-tRNA ligase activity \| \| GO:0050281 \| serine-glyoxylate transaminase \| \| GO:0005524 \| ATP binding \| \| GO:0030246 \| carbohydrate binding \| \| GO:0008483 \| transaminase activity \| \| GO:0016301 \| kinase activity \| \| GO:0016410 \| N-acyltransferase activity \| \| GO:0003723 \| RNA binding \| \| GO:0005509 \| calcium ion binding \| \| GO:0005488 \| binding \| \| GO:0032559 \| adenyl ribonucleotide binding \| \| GO:0003779 \| actin binding \| \| GO:0016740 \| transferase activity \| \| GO:0003676 \| nucleic acid binding \| | \| 0.0031 \| \| --- \| \| 0.0138 \| \| 0.0319 \| \| 0.0319 \| \| 0.0436 \| \| 0.1499 \| \| 0.0382 \| \| 0.2776 \| \| 0.2776 \| \| 0.2905 \| \| 0.3674 \| \| 0.0348 \| \| 0.053 \| \| 0.5121 \| \| 0.3163 \| \| 0.0081 \| | \| GO:0008236 \| serine-type peptidase activity \| \| --- \| --- \| \| GO:0004792 \| thiosulfate sulfurtransferase activity \| \| GO:0000062 \| fatty-acyl-CoA binding \| \| GO:0016884 \| carbon-nitrogen ligase activity, with gl... \| \| GO:0016746 \| transferase activity, transferring acyl ... \| \| GO:0003723 \| RNA binding \| \| GO:0016874 \| ligase activity \| \| GO:0016491 \| oxidoreductase activity \| \| GO:0019904 \| protein domain specific binding \| \| GO:0008270 \| zinc ion binding \| \| GO:0008233 \| peptidase activity \| \| GO:0046872 \| metal ion binding \| \| GO:0005524 \| ATP binding \| \| GO:0043169 \| cation binding \| | \| 0.001 \| \| --- \| \| 0.024 \| \| 0.072 \| \| 0.094 \| \| 0.116 \| \| 0.173 \| \| 0.092 \| \| 0.375 \| \| 0.392 \| \| 0.484 \| \| 0.016 \| \| 0.455 \| \| 0.605 \| \| 0.455 \| |
